# Variation in blood test monitoring practices for patients treated with conventional and biologic DMARDs

**DOI:** 10.1093/rap/rkaf044

**Published:** 2025-04-16

**Authors:** Joanne Ablewhite, Amy Fuller, Abdullah Almayahi, Abhishek Abhishek

**Affiliations:** Academic Rheumatology, School of Medicine, University of Nottingham, Nottingham, UK; Academic Rheumatology, School of Medicine, University of Nottingham, Nottingham, UK; Nottingham University Hospitals NHS Trust, Nottingham, UK; Academic Rheumatology, School of Medicine, University of Nottingham, Nottingham, UK

Key message

- There is substantial variation in the recommended frequency of monitoring blood tests in people treated with DMARDs.

Dear Editor, Between 11% and 35% of people prescribed DMARDs did not adhere to monitoring recommendations according to previous surveys conducted in the UK >20 years ago [1, 2]. Similar findings were reported from The Netherlands [3], while a study from Sweden reported 80% of patients with psoriasis with or without arthritis are not receiving recommended blood test monitoring while prescribed immunosuppressive treatments [4]. In recent years, the British Society for Rheumatology and other specialist societies have issued recommendations on the intervals between monitoring blood tests for the early detection of hepatic, haematological and renal adverse reactions to DMARDs [5, 6]. Whether this has standardized clinical practice in recent years has not been evaluated. We surveyed people with inflammatory conditions about the frequency of monitoring blood tests recommended to them by their clinical care team and their adherence with such recommendations.

We undertook a cross-sectional survey that was approved by the West Midlands Black Country Research Ethics Committee (Ref: 21/WM/0285). We recruited participants from rheumatology, dermatology, and gastroenterology clinics in National Health Service hospitals in Sheffield, Norwich and Nottingham and via advertisements promoted by the National Rheumatoid Arthritis Society, Crohn’s & Colitis UK, National Ankylosing Spondylitis Society, Lupus UK and Psoriasis Association. Eligible participants were required to be ≥18 years of age, diagnosed with an inflammatory condition (i.e. RA, Crohn’s disease, ulcerative colitis, psoriasis with or without arthritis, AS and SLE) and currently treated with a DMARD for ≥6 months. They self-reported age, gender, ethnicity, physician-diagnosed inflammatory condition, presence of pre-specified cardiovascular comorbidities and cardiovascular risk factors, the recommended interval between monitoring blood tests by their clinical care team and how often they did not comply with such a recommendation. Non-compliance was defined as either missing a blood test or delaying attending a blood test by >1 month in at least 2 of the previous 10 occasions. Number (%), mean (s.d.) and median [interquartile range (IQR)] were used for descriptive purposes. Data were analysed using Stata/MP (StataCorp, College Station, TX, USA).

Data for 630 eligible responders [613 (97.3%) White, 466 (74.0%) women] were included in this analysis. Their mean age was 34.36 years (s.d. 15.73). Their self-reported diagnoses were RA [*n* = 143 (22.7%)], psoriasis with or without arthritis [*n* = 113 (18.0%)], inflammatory bowel disease [*n* = 182 (28.9%)], AS [*n* = 179 (28.4%)] and lupus [*n* = 12 (1.9%)]. As shown in Fig. 1, among them, 220 (34.9%) self-reported being prescribed conventional DMARDs (cDMARDs) alone, 237 (37.6%) self-reported being prescribed biologic DMARDs (bDMARDs) or targeted synthetic DMARDs (tsDMARDs) alone and 173 (27.5%) self-reported being prescribed a combination of cDMARDs with bDMARDs or tsDMARDs. The usual place of monitoring blood tests was primary care for 335 (53.2%) participants, hospital for 264 (41.9%) participants and either primary care or hospital for 27 (4.3%) participants. Data on the location of monitoring blood tests were missing for four patients.

**Figure 1.**
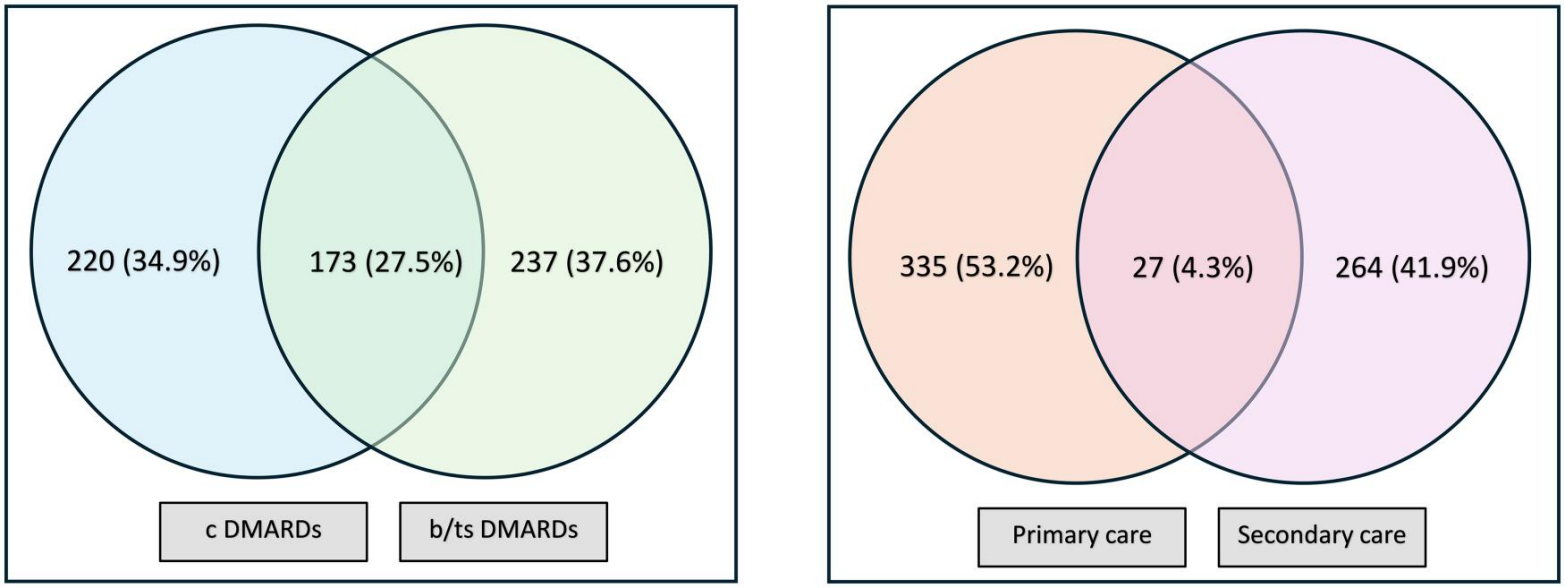
DMARD prescription and location of monitoring blood tests. Left panel: Just over one-third of people self-reported being prescribed either cDMARDs or a b/tsDMARD as monotherapy and just over one-quarter were prescribed a combination of cDMARDs and b/tsDMARDs. Right panel: Just over half of people underwent monitoring blood tests in primary care, fewer than half underwent monitoring blood tests in secondary care and a very small minority underwent monitoring blood tests in either setting

The median recommended interval between monitoring blood tests was 3  months (IQR 2–3) overall. Among those prescribed methotrexate (*n* = 203), thiopurines (*n* = 114) and leflunomide (*n* = 21), the recommended interval between monitoring blood tests was 3 (IQR 1–3), 3 (IQR 2–3) and 1 (IQR 1.5–3) months, respectively. In those prescribed bDMARD or tsDMARD monotherapy, the recommended interval between monitoring blood tests was 3 months (IQR 3–6) compared with 3 months (IQR 2–3) in those prescribed combined cDMARDs and bDMARDs or tsDMARDs. In those prescribed 5-aminosalicylic acid monotherapy (*n* = 19, 17 prescribed sulfasalazine), the median recommended interval between monitoring blood tests was 3 months (IQR 3–3).

A total of 51 (8.1%) participants missed or delayed by >1 month at least 2 of 10 previous monitoring blood test appointments. All of them were <60 years of age. This observation is limited by the fact that there were only 42 participants >60 years in age. Non-adherence was comparable in women [37 (8.0%)] and men [14 (8.5%)]. It was greater in magnitude in those prescribed bDMARDs or tsDMARDs alone [*n* = 28 (11.8%)] than in those prescribed cDMARDs alone [12 (5.5%)] and cDMARDs with bDMARDs or tsDMARDs in combination [11 (6.4%)].

In conclusion, we found substantial variations in the recommended frequency of monitoring blood tests in this large survey, suggesting the need to standardize monitoring, e.g. by developing a single national guideline issued by NICE. A limitation of this study is that we did not assess reasons for variations in recommended intervals between monitoring blood tests, e.g. due to risk factors of drug-induced toxicity, coexisting renal disease or the presence of abnormal baseline blood test results. These should be explored in future studies.

In general, patients adhered with monitoring blood tests. Adherence was lower in those <60 years of age and in those prescribed bDMARD or tsDMARD monotherapy. There were only a minority of people >62 years of age, and further research is needed in this population. These findings are limited by potential bias due to the self-reported nature of the data, which may have inflated the adherence estimates.

## Data Availability

De-identified data are available upon reasonable request to the corresponding author.
